# Depletion of β-sitosterol and enrichment of quercetin and rutin in *Cissus quadrangularis* Linn fraction enhanced osteogenic but reduced osteoclastogenic marker expression

**DOI:** 10.1186/s12906-020-02892-w

**Published:** 2020-04-03

**Authors:** Jetsada Ruangsuriya, Suporn Charumanee, Supat Jiranusornkul, Panee Sirisa-ard, Busaban Sirithunyalug, Jakkapan Sirithunyalug, Thanawat Pattananandecha, Chalermpong Saenjum

**Affiliations:** 1 Department of Biochemistry, Faculty of Medicine, Chiang Mai University, Chiang Mai, 50200 Thailand; 2 Cluster of Excellence on Biodiversity based Economic and Society (B.BES-CMU), Chiang Mai University, Chiang Mai, Thailand; 3 Department of Pharmaceutical Sciences, Faculty of Pharmacy, Chiang Mai University, Chiang Mai, 50200 Thailand

**Keywords:** *Cissus quadrangularis* Linn, Osteoporosis, Human osteoblast cell line, Ethanolic fractionation

## Abstract

**Background:**

*Cissus quadrangularis* Linn. (CQ) has been used in Indian and Thai traditional medicine for healing bone fractures because of numerous active ingredients in CQ. It is still unclear which compounds are the active ingredients for bone formation.

**Methods:**

The molecular docking technique, the ethanolic extraction along with hexane fractionation, and an in vitro experiment with a human osteoblast cell line (MG-63) were used to narrow down the active compounds, to prepare the CQ extract, and to test biological activities, respectively.

**Results:**

The molecular docking technique revealed that quercetin and β-sitosterol had highest and lowest potential to bind to estrogen receptors, respectively. Compared to the crude ethanol extract (P1), the ethanolic fraction (P2) was enriched with rutin and quercetin at 65.36 ± 0.75 and 1.06 ± 0.12 mg/g, respectively. Alkaline phosphatase (ALP) activity was significantly enhanced in osteoblasts exposed to the P2 in both tested concentrations. The amount of hydroxyproline was slightly increased in the P1 treatment, while osteocalcin was inhibited. Moreover, the P2 significantly activated osteoprotegerin (OPG) and inhibited receptor activator of nuclear factor κ ligand (RANKL) expression.

**Conclusions:**

Taken together, the enriched rutin and quercetin fraction of CQ triggered the molecules involved in bone formation and the molecules inhibiting bone resorption.

## Background

Osteoporosis is an age-related disease, which is attributed to a relatively low bone mineral density (BMD) when compared with normal individuals. This phenomenon is due to the imbalance of the bone remodeling process, in which the rate of bone resorption is much higher than that of bone formation, leading to bone loss and risk of bone fractures [1]. The primary cause of osteoporosis is the deficiency of sex steroid hormones, estrogen and/or testosterone. Even though the incidences of osteoporosis are dominantly found in women, men could be suffered from osteoporosis in similar fashions [2]. Currently, standard treatments of osteoporosis pose various side effects and shortcomings [3]. Even though novel drugs for osteoporosis treatments that act on different targets of the disease mechanisms have been developed [4], searching for medicinal plants to rectifying the imbalance of bone metabolism is also a great potential alternative. One of the potential plant candidates is *Cissus quadrangularis* Linn (CQ), which has been claimed to promote bone formation in traditional Indian medicine.

CQ, is commonly found in tropical countries and has been reported as a traditional medicinal plant for the treatment of many diseases. The stems of CQ have been reported to contain various biological activities – e.g. antioxidative as well as antimicrobial activities [5], antinociceptive potential [6], abilities to protect and heal peptic ulcers [7], induction of weight loss in obese subjects [8], properties such as an anticonvulsant, an analgesic, a smooth muscle relaxant [9], activities against hemorrhoids [10] as well as atherosclerosis [11], and acceleration of bone fracture healing [12, 13]. However, the effects of CQ on bone are more prominent than others. Ethanolic extracts of CQ supported bone formation in fetal rats when administered to the pregnant rats [14]. In addition, the ethanol extract of the CQ enhanced alkaline phosphatase (ALP) activity, a classical marker for bone formation, in murine osteoblasts occurs via the MAPK (mitogen-activated protein kinase) signaling pathway [15]. By using an osteoblastic cell line (SaOS-2) as a model, the CQ extract showed its ability to promote cellular proliferation and matrix calcification by promoting the expression of IGF-I, II and IGFBP-3 [16, 17]. Apart from the ethanolic part, petroleum ether extract of the CQ exhibited similar effect on promoting osteoblastic differentiation of rat bone marrow mesenchymal stem cells [18]. Furthermore, the studies using ovarectomised (OVX) animal models, a standard model for osteoporosis, strongly indicated the effect of CQ extract on amelioration of osteoporotic conditions in OVX mice [19] and rats [20–23], indicated by the increase in bone mass and bone mineral density. The evidence here strongly suggested that there might be certain active chemical ingredients in CQ extracts.

The varieties of chemical components in CQ have been documented. Stems of CQ contain a relatively higher amount of alkaloids and phenols than those of its roots and its leaves [24]. Calcium oxalate, cardiac glycosides, flavonoids, kaempferol, quercetin, stilbene derivatives, tannins, triterpenes (α-, β-amyrins) were also reported [25, 26]. It has further been reviewed that ascorbic acid, triterpene, β-sitosterol, ketosteroids, two asymmetrical tetracyclic triterpenoids, and calcium were the major compounds found in CQ [27]. However, it has not been ascertained which components are the actual active compounds due to the divergence of documented evidence. In this study, it was concluded from previous literature [14–17, 20, 22] that ethanolic extract of CQ might be of benefit to bone metabolism. Hence, the objective of this study was to investigate the responses of osteoblastic cell line (MG-63 cells) upon the treatment of CQ extract derived from 80% ethanol extraction, and partitioned by hexane, with the focus on active ingredients from molecular docking technique guidance.

## Methods

### Chemicals and reagents

Quercetin dihydrate **(**HWI ANALYTIK GmBH, Germany), β-sitosterol (Sigma, USA), Human sRANK-Ligand (EDK) (PREPROTECH), Human Osteocalcin Instant ELISA (eBioscience), Human Osteoprotegerin Instant ELISA (eBioscience), Quant-iT PicoGreen dsDNA Assay Kit (Invitrogen, USA).

### Molecular docking studies

**Fig. 1:**
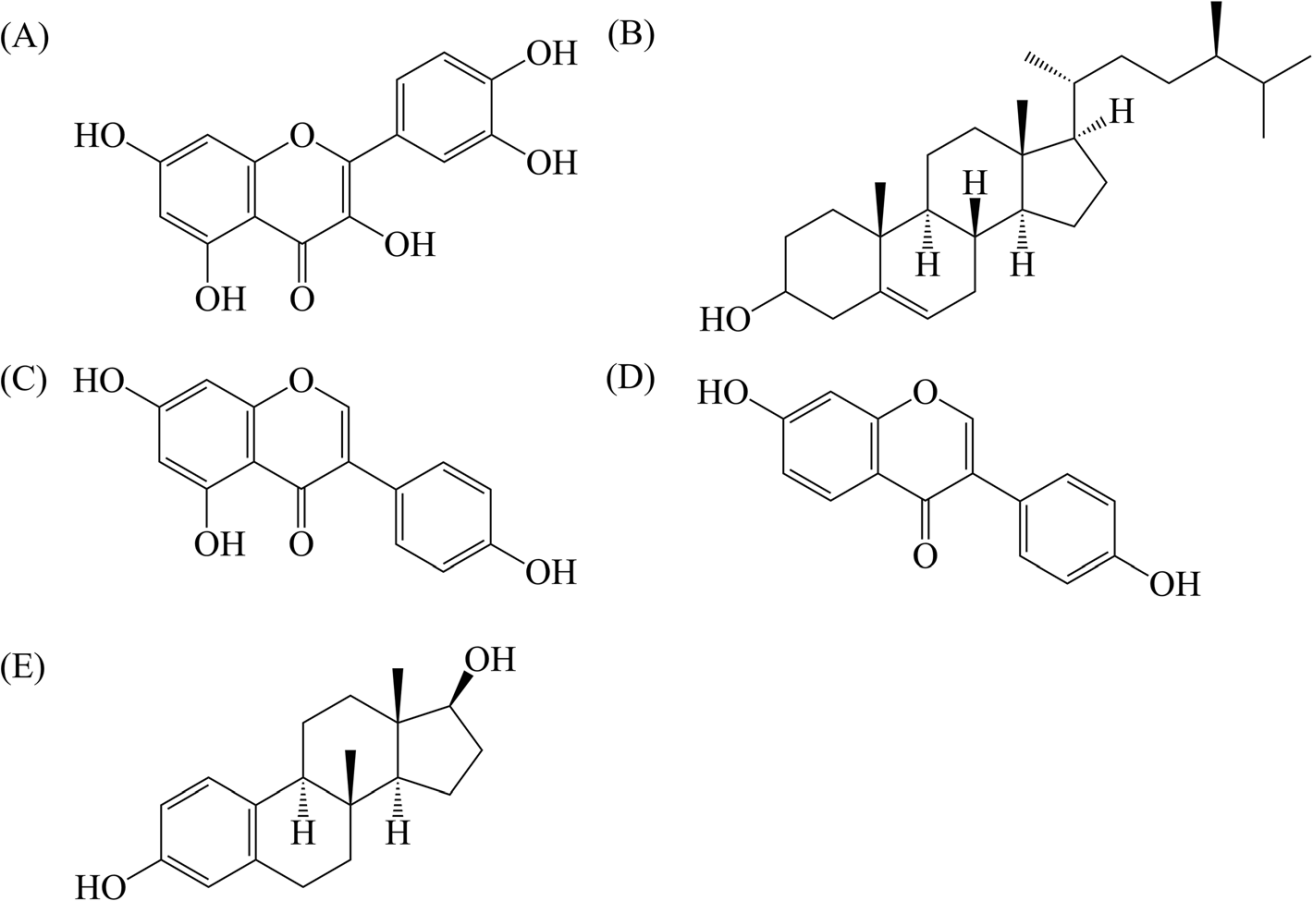
Chemical structures of candidate compounds used in the molecular docking study **a** quercetin, **b** β-sitosterol, **c** genistein, **d** diadzein, and **e** estradiol

As there are varieties of biological active compounds in CQ extract, molecular docking could narrow down the group of the compound we should focus on. Activations of estrogen receptors (ER) through those active compounds potentially activate the cellular activity and support bone health. Initially, 2D and 3D structures of two biologically active compounds in CQ extract, quercetin and β-sitosterol (Fig. 1), two known natural positive controls, genistein and diadzein, and estradiol (Fig. 1) as a physiological positive control were drawn using ChemBioOffice 2012. Then, all compounds were docked and analyzed using CDOCKER in Discover Studio version 2.5. The more the negative energy gains, the higher the affinity binds to ER.

### Preparation of CQ extract

CQ was collected from Lampang Herb Conservation, Lampang province, Thailand on December, 2013. A voucher specimen was prepared and preserved at the Faculty of Pharmacy, Chiang Mai University. An aerial part of CQ was cleaned, dried and ground into coarse powder. The powder was extracted with 80% ethanol at 80 °C. An obtained solution was separated into two equal parts for sample preparation. The first part of the solution was filtered and removed from the solvent under reduced pressure. The ultimate yields of the ethanolic extract (crude extract), which was designated as P1, were also recorded. The second part of the solution was partitioned with hexane to eliminate non-polar compounds. Only the ethanolic fraction was collected and removed from the solvent under reduced pressure. Finally, the ultimate yield of the ethanolic fraction, designated as P2, was also recorded.

The extracts were dissolved in DMSO (Dimethyl sulfoxide) and supplemented into cell culture medium containing the extracts of P1 or P2 at 50 and 100 ppm, with the final concentration of the DMSO less than 0.05% *v/v*. This set of mediums was called the “conditioned medium”.

### Determination of *β*-sitosterol, quercetin and rutin in the CQ extracts by reversed-phase HPLC

The contents of β-sitosterol, quercetin and rutin in the CQ extracts were determined by using reversed-phase HPLC (RP-HPLC) from Shimadzu system (Japan), including LC-10AV *VP* pumps and SPD-10AV *VP* with UV detector. The column for the separation was 250 × 4.6 mm in diameter (SymmetryShield RP18 C18 (Water Co, Ltd.)). Mobile phases used for determination of β-sitosterol [28], quercetin [29], and rutin [30] were acetonitrile:methanol (85:15), 5 mM KHPO_4_:acetonitrile:methanol (49:40:11), and de-ionized (DI) H_2_O:methanol:triethylamine (60:40:0.1), respectively. The flow rates and detection wavelengths for β-sitosterol, quercetin, and rutin were 1.0, 0.7, 0.5 mL/min and at the wavelength of 202, 350, 256 nm, respectively. Both of the CQ extracts, P1 and P2, were subjected to the RP-HPLC in parallel with the correlated known concentration standards, β-sitosterol (Sigma, USA), quercetin dihydrate (GmBH, Germany) and rutin (Sigma, USA). The concentrations of the three compounds were calculated from the peak areas using the calibration curves. The results were performed in triplicate.

### Culture of MG-63 cells and biochemical tests

#### Cell culture and treatments

MG-63 cells were purchased from American Type Culture Collection (ATCC, CRL-1427). The cells were cultured in Dulbecco’s modified eagle medium (DMEM) and supplemented with 10% fetal bovine serum (FBS), 1% Penicillin/Streptomycin and 1% non-essential amino acids. Upon confluence, the cells were trypsinised and plated at 1.0 × 10^4^ cells/cm^2^ into each well of 24-well plates or 96-well plates, and were performed in triplicate, per treatment condition. After 24 h of plating cells, the culture medium was replaced with the conditioned media containing different concentrations of P1 or P2. The plates were incubated at 37 °C in a humidified condition containing 5% CO_2_ for 4 and 7 d, with medium change at a 4 d interval.

Culture medium supernatants were collected, and the cells were lysed to yield cell lysates using CelLytic™ M Cell Lysis Buffer (Sigma, C2978) and stored at − 80 °C for further analyses.

#### Cytotoxicity test by WST-1 assay

MG-63 cells were plated into each well of a 96-well plate and treated with the culture media containing either P1 or P2 at different concentrations from 25 to 200 ppm for 4 d. Cell proliferation reagent WST-1 (Roche, Switzerland) was added into the treated cells and the plate was then incubated at 37 °C for 45 min. The absorbance of each well of the plate was read at 445 nm using a multimode detector (Beckman Coulter, USA). Cell viability was then calculated relative to the untreated control.

#### DNA and protein measurement

The amount of DNA was quantified by Quant-iT PicoGreen Assay (Invitrogen, P11496), according to manufacturer’s protocols. Briefly, 10 μL of the cell lysates was mixed with a working concentration of the dye, and the fluorescent data were collected at the wavelength of excitation (480 nm) and emission (530 nm) using the multimode detector. The λDNA at different concentrations was used to establish a standard curve.

The protein produced by MG-63 cells was analyzed using house-prepared Bradford reagent by adding 10 μL of the cell lysates into 90 μL of working Bradford solution. The absorbance data at 595 nm of the mixture were collected with the multimode detector. The bovine serum albumin (BSA) at different concentrations was used to establish a standard curve.

#### Quantification of hydroxyproline

The hydroxyproline was measured in the lysate by RP-HPLC according to the methods described elsewhere [31] with slight modifications. Briefly, the lysates were subjected to acid hydrolysis using 6 M HCl and measured, to obtain hydroxyproline, by RP-HPLC. The column used in this analysis was Kinetex C18 column, 250 × 4.6 mm in diameter (Phenomenex Co., Ltd.). The mobile phase consisted of 100 mM sodium acetate buffer and acetronitrile (93:7) with the flow rate of 0.3 mL/min, and the wavelength for detection was at 495 nm. Different concentrations of the standard hydroxyproline were used to set a standard curve, and the amounts of hydroxyproline in all samples were calculated accordingly.

#### Determination of alkaline phosphatase (ALP) activity

ALP activity was determined by the rate of *p*-nitrophenol phosphate (pNPP) conversion to *p*-nitrophenol (pNP). Briefly, 10 μL of the cell lysates was added into 90 μL of 1 mM pNPP in the diethanolamine buffer, pH 9.8. The mixture was mixed well and incubated at 37 °C for 75 min. The reaction was then stopped by adding 25 μL of 1 M NaOH solution into the reaction mixture, and the absorbance was read at 405 nm using the multimode detector. The standard solution of pNP at different concentrations in the buffer was used to establish a standard curve. The ALP activity was expressed in the normalized values of the total protein.

#### Detection of osteocalcin (OC), osteoprotegerin (OPG), and receptor activator of nuclear factor kappa ligand (RANKL) expression by ELISA

Commercially available ELISA kits were used to measure the expression levels of OC, OPG, and RANKL. The kits for determination of OC (BMS2020INST) and OPG (BMS2021INST) were purchased from eBioscience, while that of RANKL (900-K142) was from PreproTech. The procedures to determine the level of expression followed the manufacturer protocols, accordingly.

### Statistical analysis

One way analysis of variance (one way ANOVA), followed by Tukey tests using StatPlus version 5.8.2.0 were employed to differentiate the significant levels among the data sets with 95% confidence (*p* < 0.05).

## Results

### Molecular docking studies

**Fig. 2:**
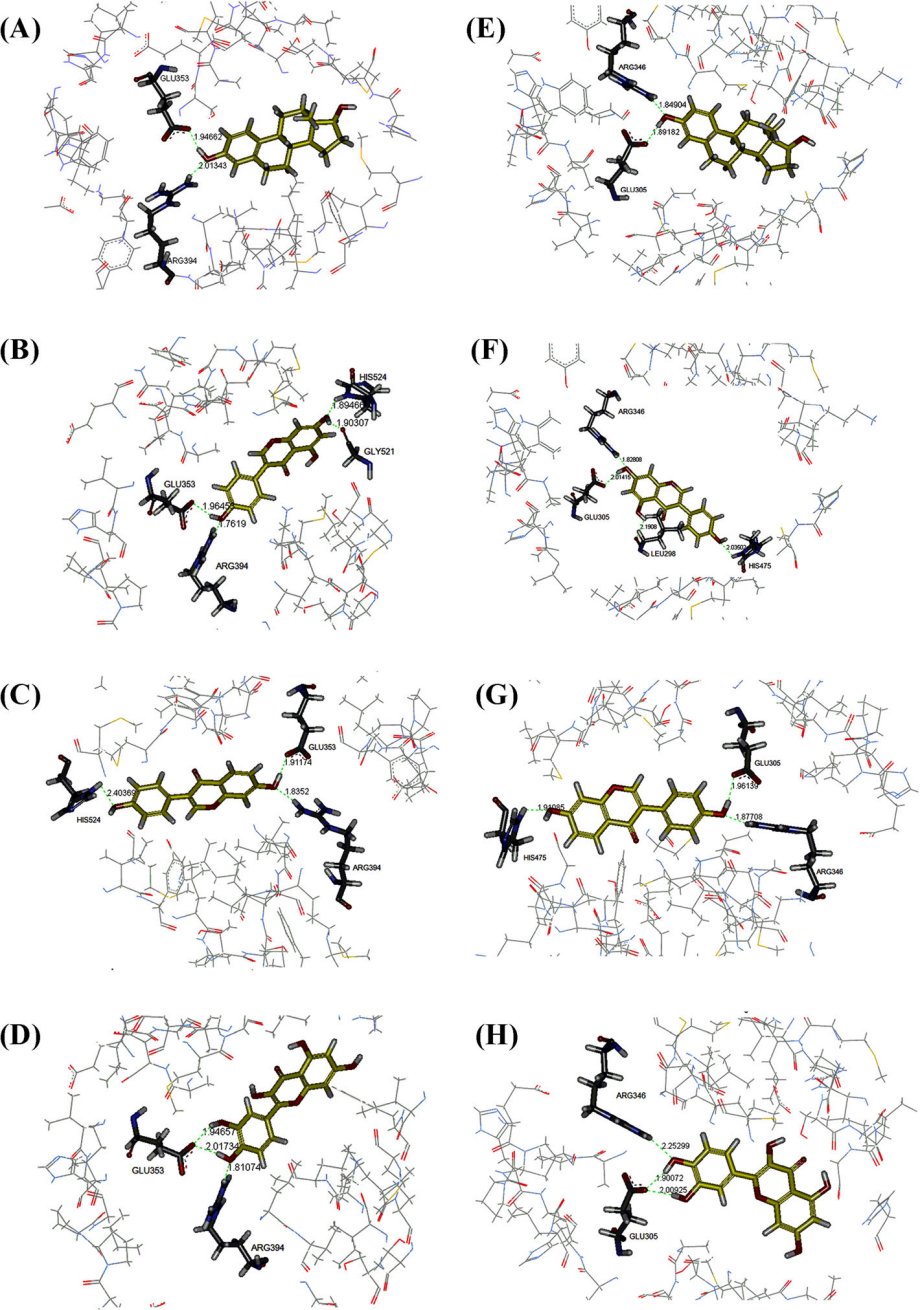
Predicted binding mode of estrogen and phytoestrogens complexed to α-ER (**a**: estradiol, **b**: genistein, **c**: diadzein, and **d**: quercetin) and β-ER estradiol (**e**: estradiol, **f**: genistein, **g**: diadzein, and **h**: quercetin)

The preliminary computerized analyses by molecular docking techniques suggested that quercetin had highest potential to bind to ER in both forms whereas β-sitosterol had the lowest capability (Table 1). Quercetin had the lowest potential energy at − 4.85 kcal/mol, the lowest differences of the two terminal hydroxyl groups at 10.8 °A, and the lowest CDOCKER energy at − 32.08 and − 37.22 kcal/mol for α-ER and β-ER, respectively. In contrast, β-sitosterol had the highest potential energy at 61.91 kcal/mol, had no two terminal hydroxyl groups, and the highest CDOCKER energy at 165.80 and 260.14 kcal/mol for α-ER and β-ER, respectively. The binding mode of estradiol, genistein, daidzein, and quercetin with their hydrogen bonding were shown in Fig. 2. According to the molecular docking results indicating the lowest binding energy of quercetin for both α-ER and β-ER, we continued to focus on the presence of quercetin in the CQ extract. In addition, rutin, a glycosylated form of quercetin, was also focused in this study.

**Table 1:** Molecular docking parameters of the active compound candidates in CQ extract

Candidate molecules	Potential energy (kcal/mol)	Distance between two terminal hydroxyl groups (°A)	CDOCKER energy (kcal/mol)	
			α-ER	β-ER
Estradiol	24.05	10.9	−11.11	−6.05
Quercetin	−4.85	10.8	−32.08	−37.22
β-sitosterol	61.97	NA	165.80	260.14
Genistein	6.69	12.2	−32.07	−35.90
Diadzein	5.84	12.2	−31.12	−34.38

### Determination of β-sitosterol, quercetin, and rutin in the CQ extracts by RP-HPLC

**Fig. 3:**
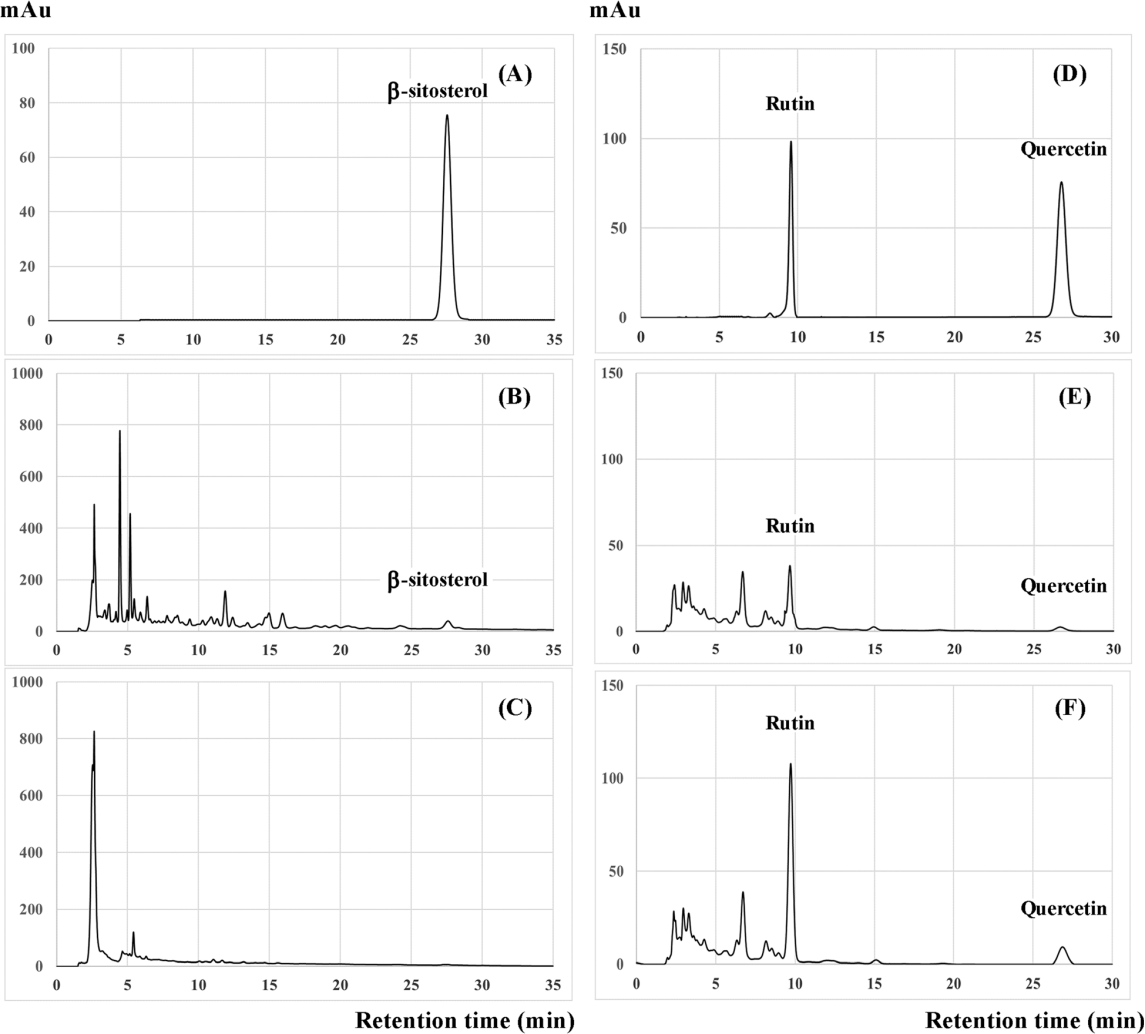
Representative chromatograms analysed by HPLC of the CQ extracts. Standard β-sitosterol was used (**a**) as the reference compound for quantification of its content in the P1 (**b**) and P2 (**c**) extracts. Likewise, standard rutin and quercetin were used (**d**) as the reference compound for the quantification of its content in the P1 (**e**) and P2 (**f**) extracts. P1 is 80% ethanol extract, whereas P2 is an ethanolic fraction

The results from RP-HPLC analyses indicated that a hexane partition completely removed β-sitosterol and enriched quercetin, as well as rutin in the CQ extract (Fig. 3). The crude ethanolic extract (P1) contained β-sitosterol 2.45 ± 0.27 mg/g, quercetin 0.97 ± 0.14 mg/g, and rutin 30.41 ± 0.24 mg/g. After a hexane partition, the ethanolic fraction (P2) contained an undetectable amount of β-sitosterol, a stable amount of quercetin 1.06 ± 0.12 mg/g and an increasing amount of rutin 65.36 ± 0.75 mg/g.

### Cytotoxicity, DNA contents, and protein production of MG-63 exposed to P1 and P2

**Fig. 4:**
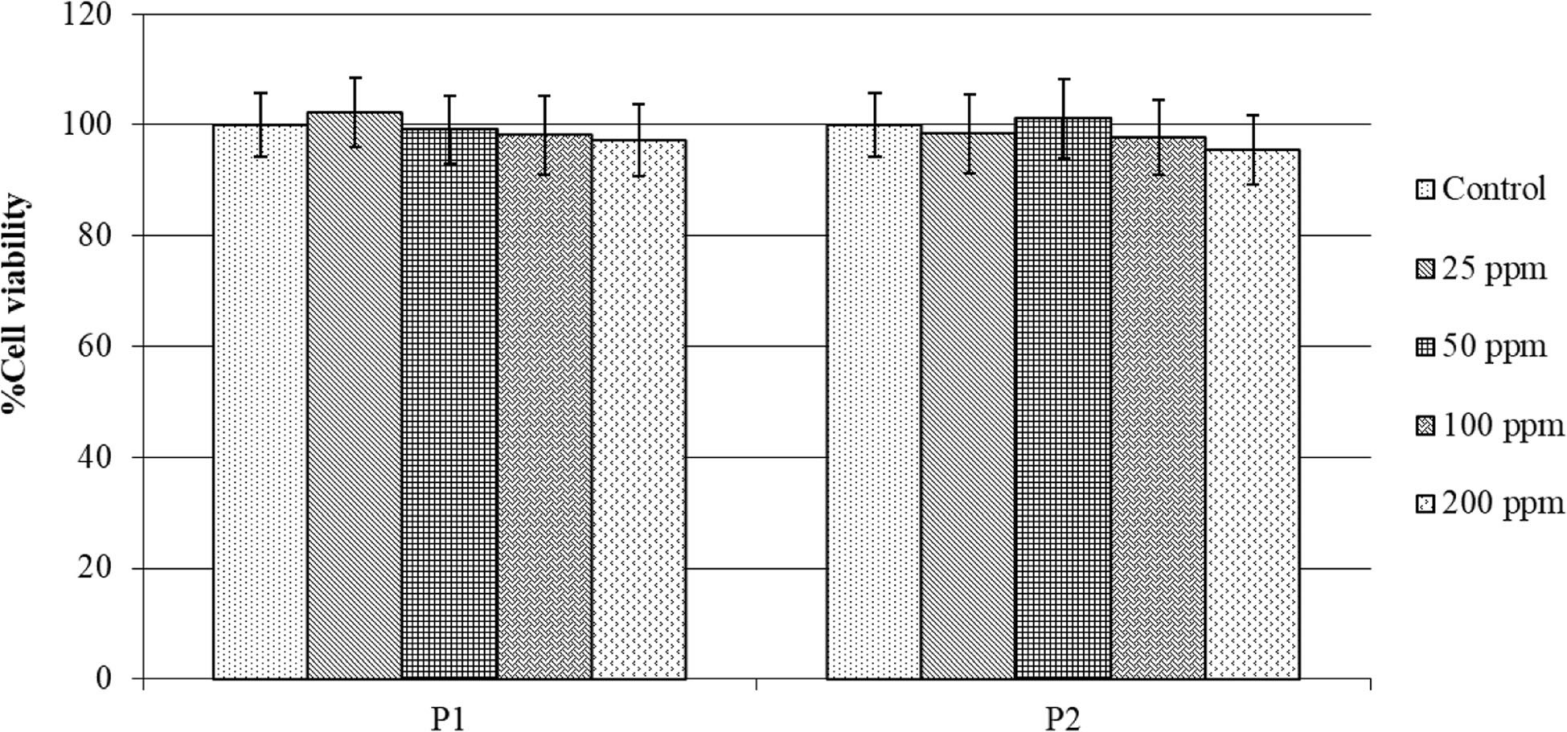
Cell viability of MG-63 cells grown in medium without (Control) or with CQ extracts (P1, P2). P1 is 80% ethanol extracts whereas P2 is an ethanolic fraction at a concentration range of 25–200 ppm

**Fig. 5:**
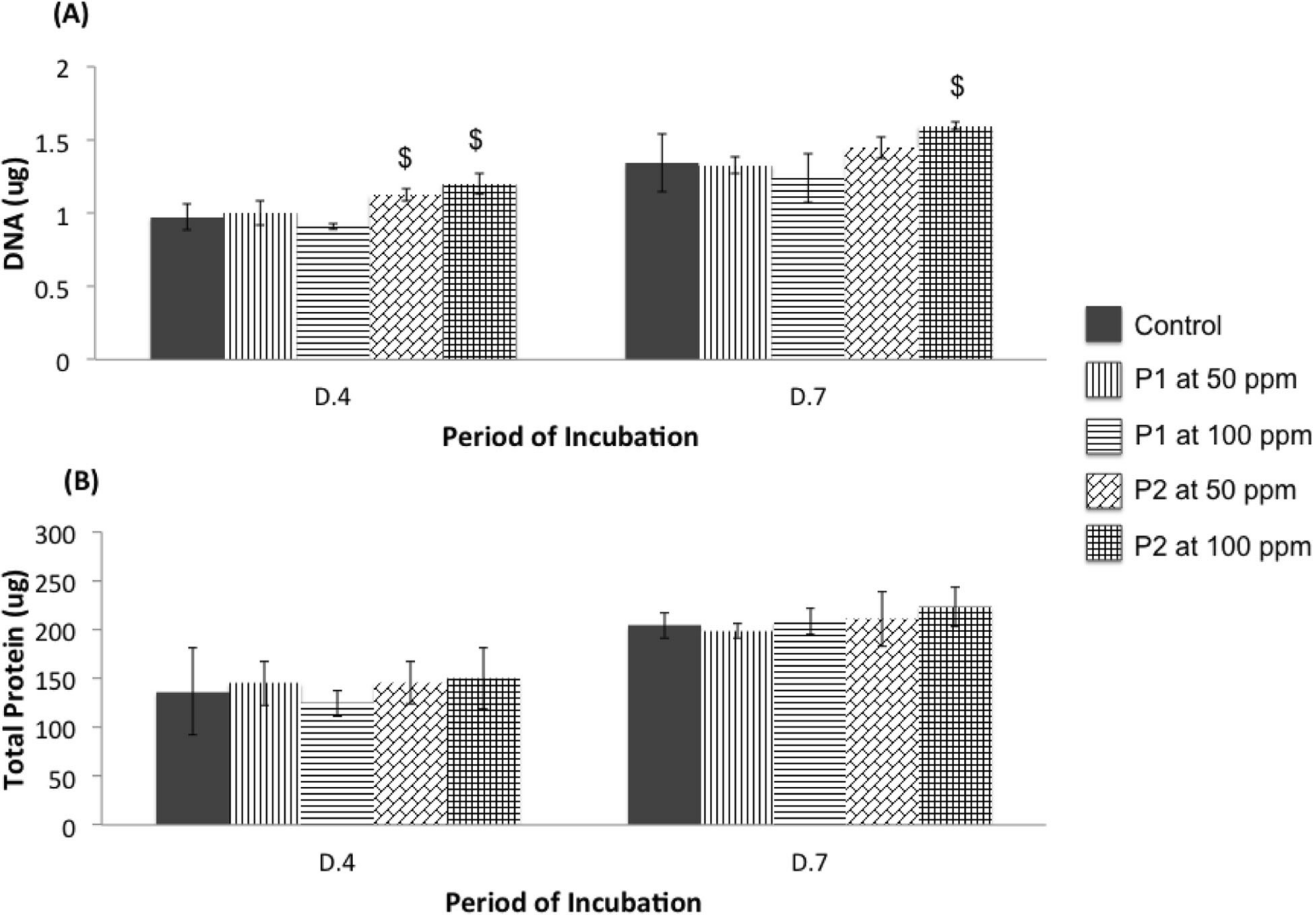
Level of DNA (**a**) and total protein (**b**) of MG-63 cells grown in medium without (Control) or with CQ extracts (P1, P2). P1 is 80% ethanol extracts, whereas P2 is an ethanolic fraction at 50 and 100 ppm. Values are mean ± SD where *n* = 6 ($ is a significant levels at *p* < 0.05 when compared to Control, P1 at 50 ppm, and P1 at 100 ppm)

P2 significantly increased the amount of DNA in MG-63, but did not affect total protein production and cell viability. The viability of MG-63 cells on the exposure of both P1 and P2 at a concentration range of 25–200 ppm remained the same as that of the control (Fig. 4). The amount of the DNA, which indicates the ability of the cells to proliferate, clearly showed that P2 significantly activated cell proliferation at both time points, especially at 100 ppm in its concentration (Fig. 5a). In contrast, P1 had no effect on cell proliferation. However, both P1 and P2 did not alter the ability of the cells to produce total proteins (Fig. 5b).

### Expression of markers indicating bone formation

**Fig. 6:**
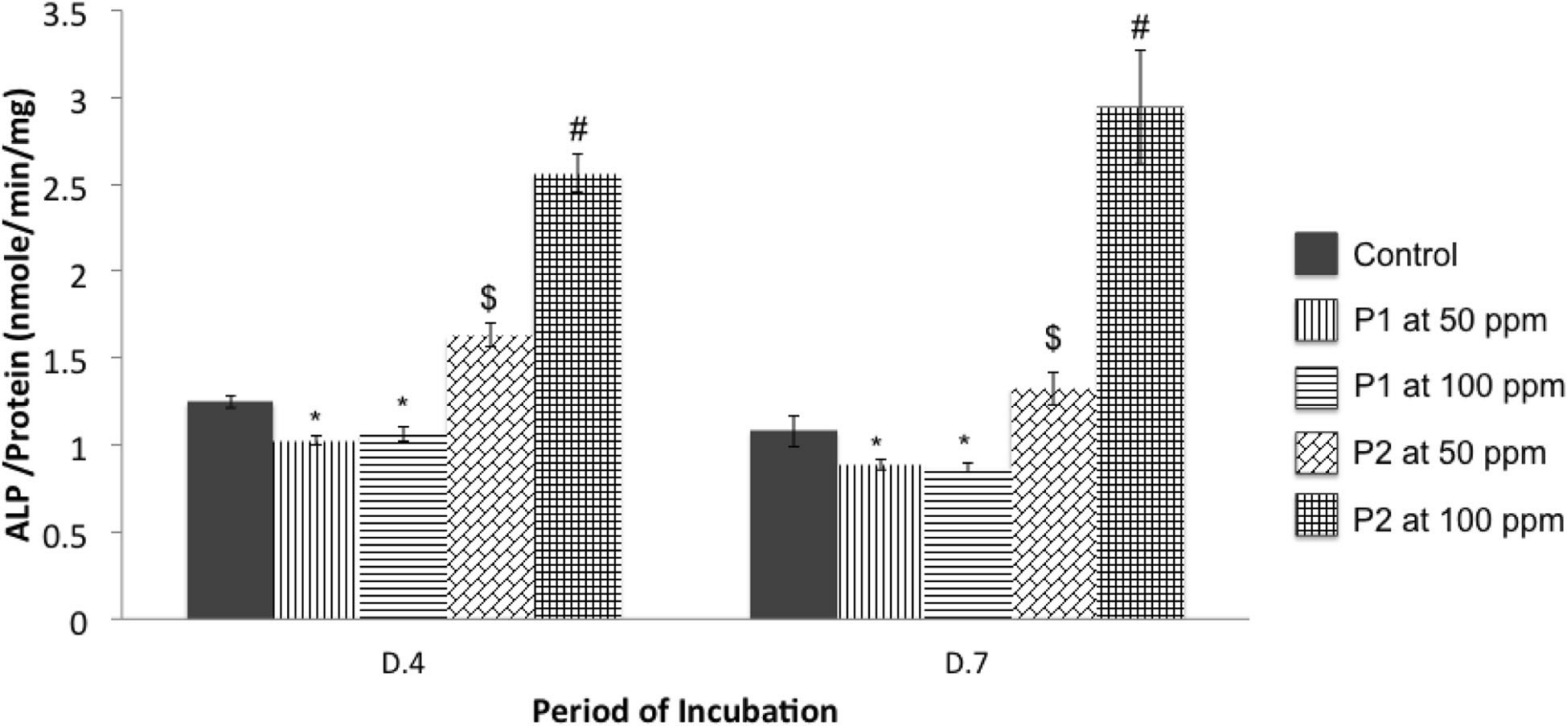
The activity of alkaline phosphatase (ALP) normalized with total protein from MG-63 cells grown in medium without (Control) or with CQ extracts (P1, P2). P1 is 80% ethanol extracts whereas P2 is an ethanolic fraction at 50 and 100 ppm. Values are mean ± SD where *n* = 6 (#, $, and * are a significant difference at *p* < 0.05; # compared to Control, P1 at 50 ppm, P1 at 100 ppm, and P2 at 50 ppm, $ compared to Control, P1 at 50 ppm, and P1 at 100 ppm, and * compared to Control)

P2 significantly activated the activity of ALP, which increased the level of collagen, but reduced the expression of OC. It was clearly shown that P2 at both 50 and 100 ppm significantly enhanced ALP activity of MG-63 cells in both time points. In contrast, P1 at both 50 and 100 ppm significantly inhibited the activity of ALP (Fig. 6).

**Fig. 7:**
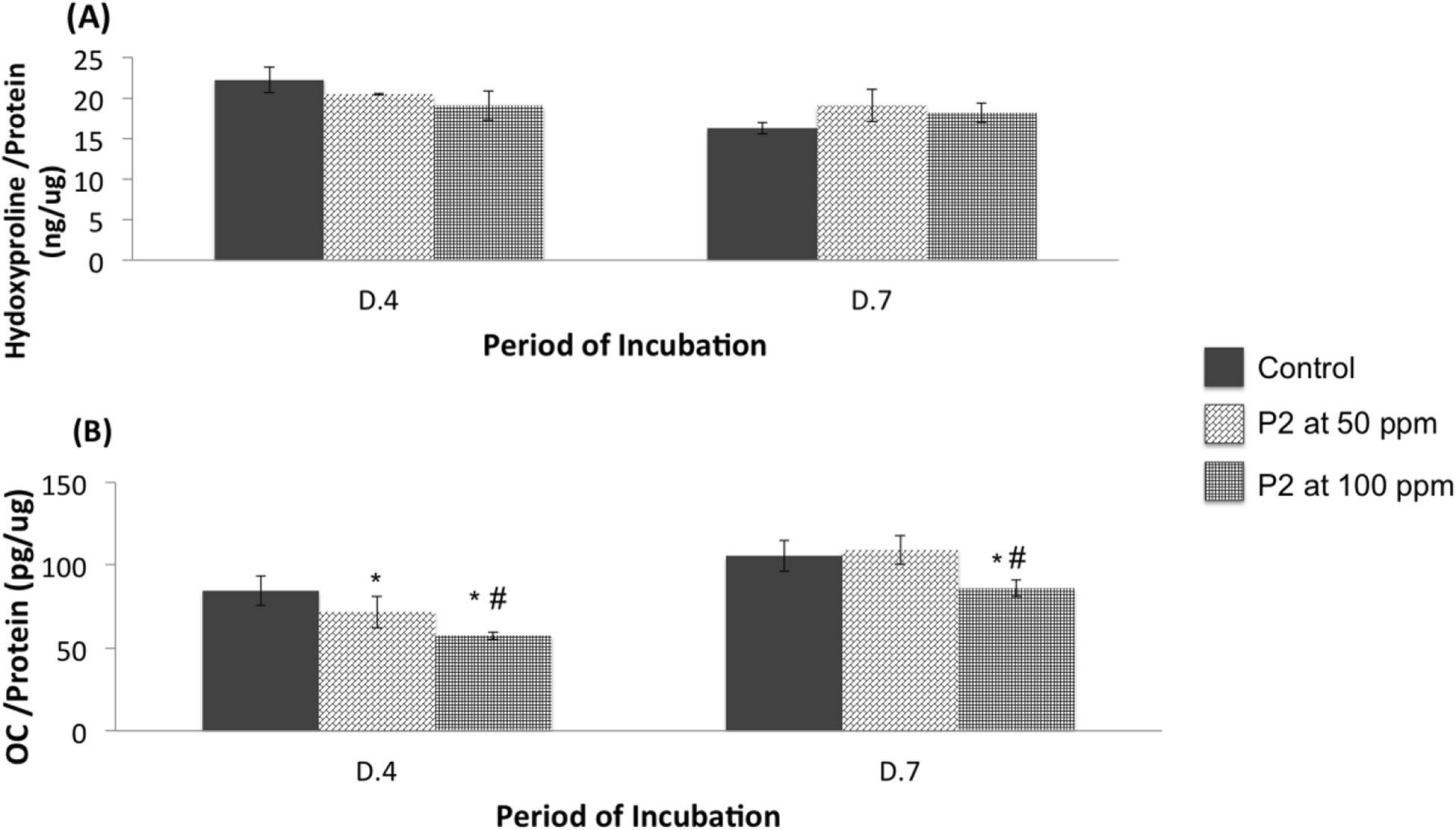
The levels of hydroxyproline (**a**) and osteocalcin (OC) (**b**) normalized with total protein from MG-63 cells grown in medium without (Control) or with CQ extracts (P2) at the concentration of 50 or 100 ppm. Values are mean ± SD where *n* = 6 (# and * are a significant difference at *p* < 0.05 compared to P2 at 50 ppm and Control, respectively)

The amount of collagen production determined by levels of hydroxyproline in the cell lysates indicated that P2 slightly increased collagen production, especially on day 7 (Fig. 7a). In contrast, the levels of OC were significantly lower in P2 supplementation, (Fig. 7b).

### Expression of markers indicating bone resorption

**Fig. 8:**
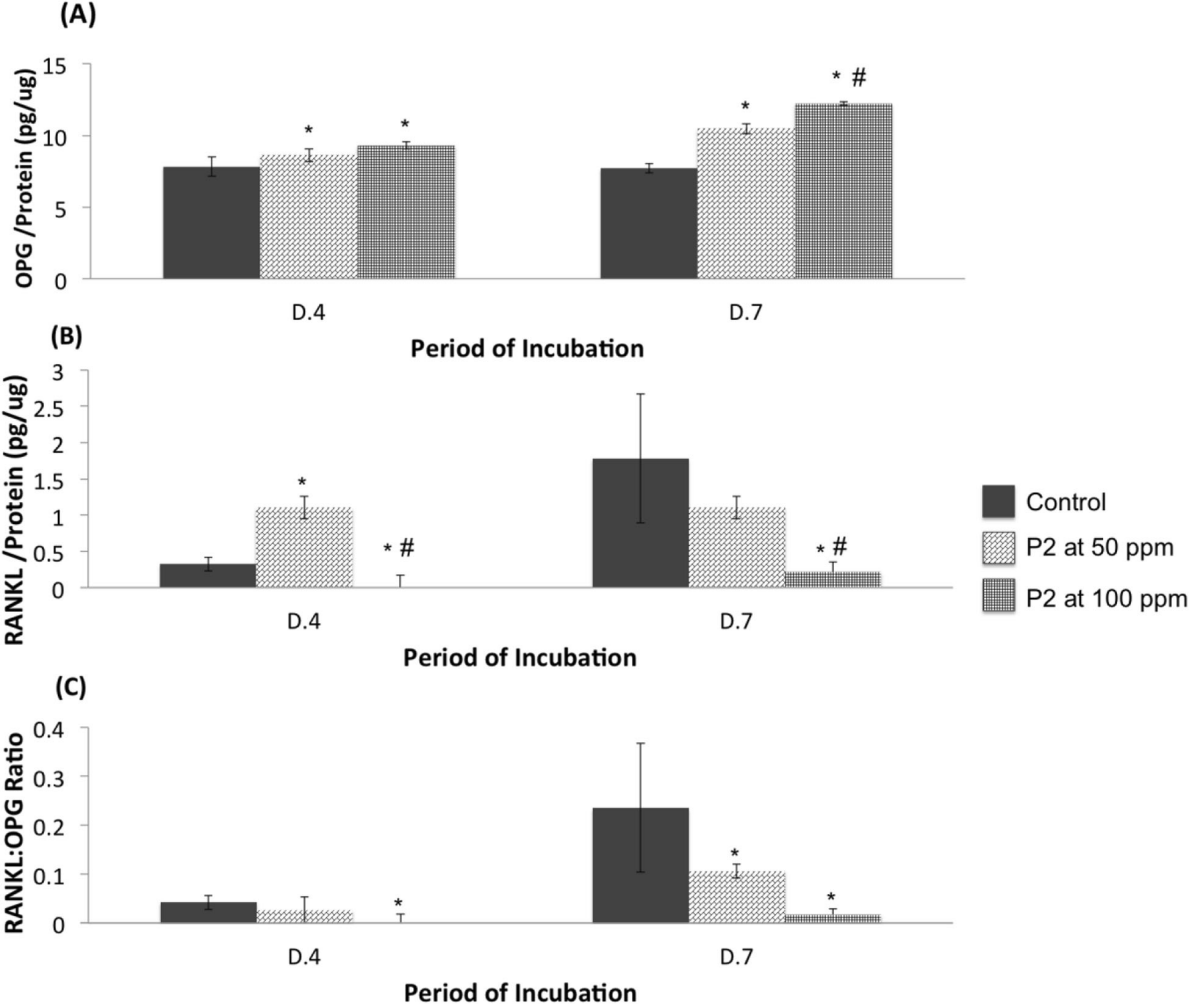
The levels of osteoprotegerin (OPG) (**a**), receptor activator of nuclear factor kappa ligand (RANKL) (**b**), and RANKL:OPG ratio (**c**) from MG-63 cells grown in medium without (Control) or with CQ extracts (P2) at the concentration of 50 or 100 ppm. Values are mean ± SD where *n* = 6 (# and * are a significant difference at *p* < 0.05 compared to P2 at 50 ppm and Control, respectively)

P2 had a strong effect on the expression of both OPG and RANKL in MG-63 cells. It was obvious that P2 statistically elevated the expression of OPG at both time points (Fig. 8a), and reduced the expression of RANKL production, especially at the concentration of 100 ppm (Fig. 8b). This result led to the significant reduction of the RANKL/OPG ratio (Fig. 8c) indicating the reduction of bone resorption.

### Activation of ALP activity by standard quercetin and rutin

**Fig. 9:**
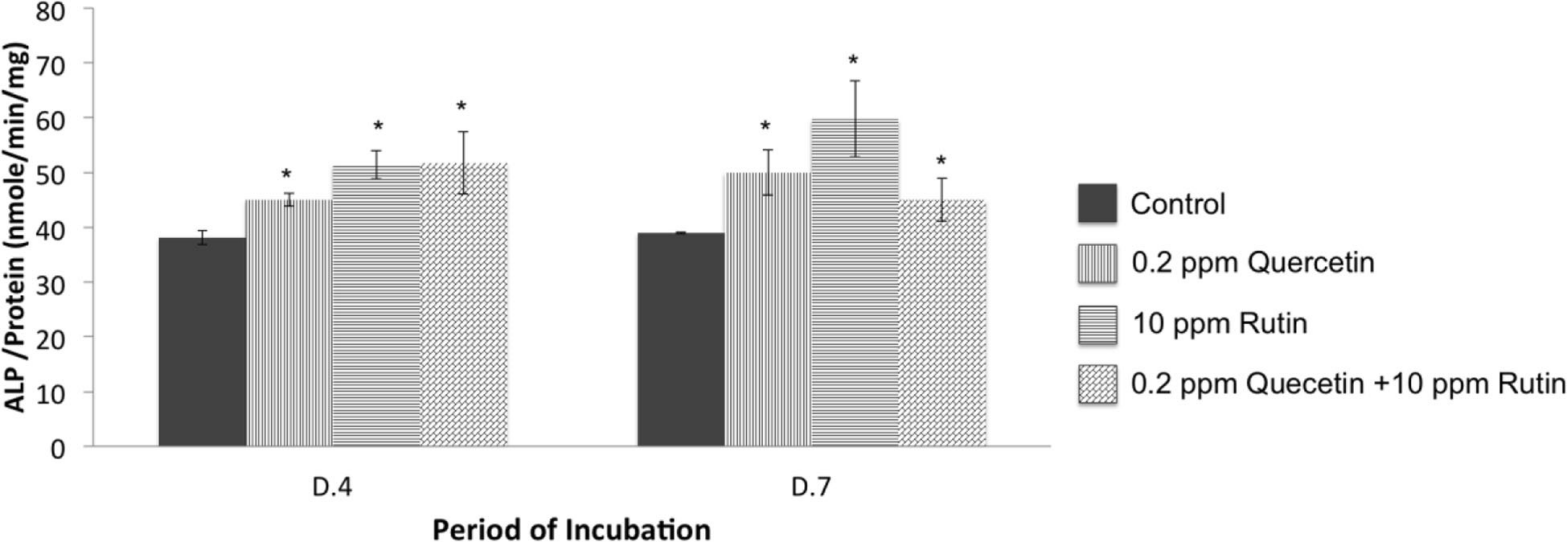
The activity of alkaline phosphatase (ALP) normalized with total protein from MG-63 cells grown in medium without (Control) or with 0.2 ppm standard quercetin, 10 ppm standard rutin, or a combination of both quercetin and rutin (0.2 ppm quercetin+ 10 ppm rutin). Values are mean ± SD where *n* = 3 (* is a significant difference at *p* < 0.05 compared to Control)

The presence of either quercetin or rutin had a positive effect on MG-63 cells by activating ALP activity (Fig. 9). The quercetin, the rutin, or a combination at a concentration equivalent to the CQ extract, significantly enhanced the activity of the ALP enzyme at both time points. This result suggested that quercetin and rutin were active compounds of CQ extracts upon activation of ALP activity.

## Discussion

The present study reported the consistent findings between molecular docking and in vitro experiments to highlight the effect of an ethanolic fraction (P2) of CQ after a hexane partition on an osteoblast cell line (MG-63). The partition process successfully eliminated all β-sitosterol in the primary ethanol extract (P1). The P2 extract significantly promoted cell proliferation, ALP activity, and OPG expression, but suppressed RANKL expression. Although the hexane extract of the CQ has been reported to posses osteoblastogenesis-promoting activities observed by the activation of ALP activity [32], our results found that the removal of the hexane soluble fraction, like β-sitosterol, provided greater benefits to osteoblast cells. The evidence regarding direct effects of β-sitosterol on osteoblasts are limited. It has been documented that β-sitosterol extracted from *Rubus chingii* did not activate ALP activity [33]. Besides, β-sitosterol, at as low of a concentration as 16 μM, potentially caused cytotoxicity in breast cancer cells [34] and a leukemic cell line [35]. It is obvious in this study that both P1 and P2, at the maximal concentration of 100 ppm, did not cause cytotoxicity to the MG-63 cells. Our result was opposite to the recent finding that 100 ppm ethanol extract of CQ significantly induced apoptosis, by activation of ROS (reactive oxygen species) formation and DNA fragmentation in A431, human epidermoid carcinoma cell line [36]. Moreover, the biphasic properties of the ethanolic extract of CQ were dependent on the concentration by which the extract at 75 and 100 ppm impaired osteoblasts viability, proliferation, and minernalisation [37]. However, the in vivo model supported the use of the ethanol extracts, without partition, at a high dose of up to 500 mg/kg, which caused no toxicity to the tested animals [20–23].

The presences of β-sitosterol in P1 at 50 and 100 ppm were equivalent to the β-sitosterol alone at 0.3 and 0.6 μM, respectively. Our study added that low concentrations of β-sitosterol had the inhibitory effect on the osteoblastic cell line used in this study. However, various concentrations of the β-sitosterol documented in publications have been reported in wide range from 0.1 to 200 μM [38, 39]. One possible explanation why our low concentrations of the β-sitosterol in the P1 fraction of the CQ exhibited the inhibitory effects on the cells might be synergistic mechanisms with of other unknown bioactive compounds in the extract [40].

P2 fraction enhanced markers, indicating bone formation, including ALP activity and hydroxyproline levels. The presence of hexane-soluble compounds, like β-sitosterol, in P1 might have a strong effect on suppression of ALP activity, whereas the absence of the hexane-soluble compounds in P2 significantly activated ALP activity. ALP has been used as a biomarker of bone formation, in combination with other biochemical markers, detectable in serum and/or urine like terminal fragment of pro-collagen and OC [41]. Elevation of these markers indicates the process of bone formation. However, P2 fraction had no effect on the level of OC, the indicator of the bone turnover process, which includes both of the bone resorption and bone formation processes [42]. The elevation of OC indicates the high rate of bone resorption as well as bone formation [43, 44]. The enrichment with quercetin and rutin in P2 significantly reduced RANKL, but enhanced OPG levels, providing a negative effect on bone resorption processes. RANKL and OPG, mainly produced from osteoblasts, activates and inhibits osteoclastogenesis, respectively. The levels of RANKL and OPG were well regulated by various biological mechanisms to maintain bone metabolism homeostasis. The ratio between RANKL and OPG is used to compare levels of osteoclastogenesis among experimental conditions, and the increase in RANKL:OPG ratio indicate the trend to support osteoclastogensis, in vice versa [45, 46].

According to the results, P2 fraction, which was enriched with rutin and quercetin, showed positive effects on bone formation. Various studies of rutin and quercetin on the support of bone formation reported that quercetin promoted osteogenesis in osteoblast cell culture model [47] and animal model [48], indicated by the increase in bone formation markers. Moreover, quercetin enhanced the osteogenic differentiation process from the progenitor stem cells [49]. In contrast, rutin had indirect support of bone formation due to the finding that it strongly increased RANKL expression in osteoblasts [50]. The effect of rutin has also been investigated in osteoclast progenitor cells, and it was found that rutin decreased ROS by deactivation of the transcription factor of NF-κB [51], resulting in the low level of RANK in osteoclast progenitor [52]. However, these reports used the pure compound of either rutin or quercetin at different concentrations. There is very limited evidence on osteogenic responses to the combination of both rutin and quercetin. We had also investigated the effect of the pure compound of quercetin and rutin on MG-cells. The presence of quercetin and rutin, either on its own or in combination, activated the activity of ALP, implying the support of bone formation. It is indicating that the P2 fraction of the CQ extract, rich in rutin and quercetin, had an ultimate benefit to osteoporotic patients, and the combination requires further studies.

## Conclusion

The ethanolic fraction P2 of CQ extract, which is relatively enriched with both rutin and quercetin when compared to the P1 fraction, has positive effects on bone health. The markers indicating bone formation, such as ALP and collagen by hydroxyproline levels, were markedly increased when exposed to P2. In addition to the significant decrease in the markers indicating bone resorption, RANKL:OPG ratio was also observed in P2 treated cells. It could be suggested that the P2 fraction of the CQ extract might be potentially developed as a therapeutic agent for osteoporosis because of the biphasic effects (supports bone formation and inhibits bone resorption).

## Data Availability

The supporting materials can be obtained upon request via email to the corresponding author.

## Abbreviations

ALP: Alkaline phosphatase
BMD: Bone mineral density
BSA: Bovine serum albumin
CQ: *Cissus quadrangularis* Linn
DI: De-ionized
DMEM: Dulbecco’s modified eagle medium
DMSO: Dimethyl sulfoxide
ER: Estrogen receptors
FBS: Fetal bovine serum
MAPK: Mitogen-activated protein kinase
OC: Osteocalcin
OPG: Osteoprotegerin
OVX: Ovarectomised
pNP: *p*-nitrophenol
pNPP: *p*-nitrophenol phosphate
RANKL: Receptor activator of nuclear factor kappa ligand
ROS: Reactive oxygen species
RP-HPLC: Reversed-phase High-performance liquid chromatography
